# Supplementary material for: Improving Machine Learning Diabetes Prediction Models for the Utmost Clinical Effectiveness
Source: J Pers Med. 2022 Nov 14;12(11):1899. doi: 10.3390/jpm12111899 (PMC9698354; doi:10.3390/jpm12111899)
Supplement: Supplementary file 1 [file jpm-12-01899-s001.zip › jpm-1954553-Supplementary.pdf]

Supplementary Materials:

Improving machine learning diabetes prediction models for the utmost clinical effectiveness

Juyoung Shin, Joonyub Lee, Taehoon Ko, Kanghyuck Lee, Yera Choi, Hun-Sung Kim

Table S1. Diabetes prediction modelling candidate variables

	**Total 112 variables**
**Physical examination**	Age, sex
	Weight, height, BMI
	Ideal body weight
	Muscle mass, fat mass
	Body fat percent
	Waist-hip ratio, waist circumference
	Systolic BP, diastolic BP, pulse rate
	FVC, FEV1, FEV/FVC, FEF 25-75%, PEFR
**Laboratory findings**	Fasting glucose, HbA1c
	total cholesterol, triglyceride, HDL-C, LDL-C
	Hemoglobin, hematocrit
	Total protein, albumin
	Uric acid
	AST, ALT
	Alkaline phosphatase, amylase, ɤ-GTP
	Globulin, albumin/globulin ratio
	BUN, creatinine
	Sodium, potassium, chloride
	calcium, phosphorus
	TSH, free T4
**Family history**	Cardiovascular/cerebrovascular disease,
	diabetes mellitus, hypertension, dyslipidemia, hepatic disease,
	thyroid diseases, rheumatoid diseases, pulmonary diseases,
	osteoporosis
	cancer
**Past history**	Cardiovascular/cerebrovascular disease,
	diabetes mellitus, hypertension, dyslipidemia, hepatobiliary disease,
	thyroid diseases, rheumatoid diseases, osteoporosis
	pulmonary diseases, renal diseases,
	gynecologic/urologic diseases, ophthalmologic diseases
	cancer

BMI, body mass index; BP, blood pressure; FVC, forced vital capacity; FEV1, forced expiratory volume in 1 second; FEF25-75, forced expiratory flow at 25% and 75% of vital capacity; PEFR, peak expiratory flow rate; HbA1c, glycosylated hemoglobin; HDL-C, high-density lipoprotein cholesterol; LDL-C, low-density lipoprotein cholesterol; AST, aspartate transaminase; ALT, alanine aminotransferase; ɤ-GTP, ɤ-glutamyl transpeptidase; BUN, blood urea nitrogen; TSH, thyroid stimulating hormone; free T4, free thyroxine.

Table S2. Characteristics of the subjects in test data

	All (n=7,676)	Incident diabetes (n=304)	No diabetes (n=7,372)
**Age, years**	45 ± 10	51 ± 9	45 ± 10
**Sex, female (%)**	3,389 (44.2)	76 (25.0)	3,313 (44.9)
**BMI, kg/m2**	23.4 ± 3.2	25.7 ± 3.1	23.3 ± 3.1
**Body fat percent, %**	26.1 ± 6.1	27.8 ± 6.3	26.1 ± 6.0
**Waist-hip ratio**	0.88 ± 0.05	0.92 ± 0.04	0.88 ± 0.05
**SBP, mmHg**	119 ± 14	125 ± 13	118 ± 14
**DBP, mmHg**	73 ± 10	77 ± 10	73 ± 10
**Laboratory finding**			
FBG, mg/dL	91 ± 12	110 ± 24	90 ± 10
HbA1c, %	5.4 ± 0.3	6.0 ± 0.3	5.4 ± 0.3
Total cholesterol, mg/dL	196 ± 34	200 ± 41	196 ± 33
Triglyceride, mg/dL	112 ± 82	163 ± 126	110 ± 79
HDL-C, mg/dL	54 ± 13	47 ± 11	54 ± 13
LDL-C, mg/dL	119 ± 30	122 ± 35	118 ± 30
AST, IU/L	23 ± 13	30 ± 18	23 ± 12
ALT, IU/L	25 ± 19	39 ± 28	25 ± 18
ɤ-GTP, IU/L	35 ± 40	63 ± 69	34 ± 38
Amylase, IU/L	86 ± 30	78 ± 25	87 ± 30
BUN, mg/dL	13 ± 4	15 ± 4	13 ± 4
Creatinine, mg/dL	0.9 ± 0.2	0.9 ± 0.2	0.9 ± 0.2
Sodium, mEq/L	142 ± 2	142 ± 2	142 ± 2
Potassium, mEq/L	4.2 ± 0.3	4.2 ± 0.3	4.2 ± 0.3
Calcium, mg/dL	9.1 ± 0.4	9.2 ± 0.4	9.1 ± 0.4
Phosphate, mg/dL	3.5 ± 0.5	3.5 ± 0.5	3.5 ± 0.5
Uric acid, mg/dL	5.4 ± 1.4	6.0 ± 1.5	5.4 ± 1.4
TSH, uIU/ml	2.26 ± 5.08	2.02 ± 1.78	2.27 ± 5.17
Hemoglobin, g/dL	14.4 ± 1.6	14.9 ± 1.4	14.4 ± 1.6
**Pulmonary function test**			
FVC, %	92.9 ± 11.0	91.9 ± 11.1	92.9 ± 11.0
FEV1, %	98.7 ± 13.0	98.4 ± 13.7	98.8 ± 13.0
FEV1/FVC, %	82.3 ± 6.7	80.3 ± 6.2	82.4 ± 6.7
FEF25-75, %	100.1 ± 26.8	96.8 ± 28.0	100.3 ± 26.8
PEFR, L/s	99.0 ± 17.5	98.3 ± 17.9	99.0 ± 17.5
**Personal history**			
Hypertension (%)	834 (11.0)	86 (28.3)	748 (10.3)
Cardiovascular diseases (%)	118 (1.6)	18 (6.0)	100 (1.4)
Cerebrovascular diseases (%)	108 (1.4)	10 (3.3)	98 (1.4)
**Family history**			
Hypertension (%)	3,166 (41.7)	156 (51.3)	3,010 (41.3)
Diabetes (%)	2,206 (29.0)	145 (47.7)	2,061 (28.3)
Cardiovascular diseases (%)	1,448 (19.1)	57 (18.8)	1,391 (19.1)
Cerebrovascular diseases (%)	1,724 (22.7)	81 (26.6)	1,643 (22.5)
**Follow-up, year**	3.9 ± 2.5	5.3 ± 2.5	3.9 ± 2.4
**Checkup, n**	3.7 ± 2.1	4.7 ± 2.3	367 ± 2.0

Categorical variables are reported as frequencies (%), and continuous variables are reported as mean ± SD.

BMI, body mass index; SBP, systolic blood pressure; DBP, diastolic blood pressure; FBG, fasting blood glucose; HbA1c, glycosylated hemoglobin; HDL-C, high-density lipoprotein cholesterol; LDL-C, low-density lipoprotein cholesterol; AST, aspartate transaminase; ALT, alanine aminotransferase; ɤ-GTP, glutamyl transpeptidase; BUN, blood urea nitrogen; TSH, thyroid stimulating hormone; FVC, forced vital capacity; FEV1, forced expiratory volume in 1 s; FEF25-75, forced expiratory flow at 25% and 75% of vital capacity; PEFR, peak expiratory flow rate
